# A large population of cell-specific action potential models replicating fluorescence recordings of voltage in rabbit ventricular myocytes

**DOI:** 10.1098/rsos.241539

**Published:** 2025-03-26

**Authors:** Radostin D. Simitev, Rebecca J. Gilchrist, Zhechao Yang, Rachel C. Myles, Francis L. Burton, Godfrey L. Smith

**Affiliations:** ^1^ School of Mathematics and Statistics, University of Glasgow, Glasgow, UK; ^2^ School of Cardiovascular and Metabolic Health, University of Glasgow, Glasgow, UK

**Keywords:** cellular excitability, rabbit ventricular myocytes, fluorescence voltage measurements, action potential waveform, parameter estimation in differential equations, noisy time series

## Abstract

Recent high-throughput experiments unveil substantial electrophysiological diversity among uncoupled healthy myocytes under identical conditions. To quantify inter-cell variability, the values of a subset of the parameters in a well-regarded mathematical model of the action potential of rabbit ventricular myocytes are estimated from fluorescence voltage measurements of a large number of cells. Statistical inference yields a population of nearly 1200 cell-specific model variants that, on a population-level replicate experimentally measured biomarker ranges and distributions, and in contrast to earlier studies, also match experimental biomarker values on a cell-by-cell basis. This model population may be regarded as a random sample from the phenotype of healthy rabbit ventricular myocytes. Univariate and bivariate joint marginal distributions of the estimated parameters are presented, and the parameter dependencies of several commonly used electrophysiological biomarkers are determined. Parameter values are weakly correlated, while summary metrics such as the action potential duration are not strongly dependent on any single electrophysiological characteristic of the myocyte. Our results demonstrate the feasibility of accurately and efficiently fitting entire action potential waveforms at scale.

## 1. Introduction

### 1.1. Cellular variability

Genetically identical cardiomyocytes, even ones that have developed in identical extracellular conditions, exhibit differences in their electrophysiological properties. Inter-cell variability has been confirmed using a range of experimental techniques in cardiac tissues from various species [1–6]. Inter-cell variability affects physiological function upstream at tissue and organ levels with one clinically important effect being that a drug therapy designed to inhibit specific ion channel(s) will have different outcomes on different members of the population [7,8]. Experimental determination of the differences in multiple ionic conductances underlying this inter-cell heterogeneity is not feasible and so it is necessary to examine them in mechanistic mathematical models.

### 1.2. Mainstream approaches to modelling of variability

Advances in the mathematical and statistical modelling of cardiomyocyte variability are summarized in [9–11], with two main strategies emerging: a ‘population-based’ approach and a ‘sample-specific’ approach [9]. Both begin with an appropriate generic cardiomyocyte action potential (AP) model as a baseline. Generic models for various species and cardiac tissues have been extensively developed over the past 70 years [12,13]. Population-based approaches proceed to generate model variants of their baseline model by randomly sampling parameter values from hypothetical distributions. In some studies, further rejection-subsampling is performed to fit presumed or experimental distributions of one or more action potential characteristics (biomarkers). Examples include the works [3,14–16]. Sample-specific modelling approaches re-estimate the parameter values of their baseline model using cell-specific datasets. Examples include the studies [17–22]. Population-based approaches offer the advantage of statistically meaningful population sizes of the order of $10^{4}$ randomized models. Such sizes are larger than the number of cells easily measurable in experiments, yield more accurate descriptive statistics and facilitate detection of effects that may go unnoticed in small-size populations. The critical drawback of population-based approaches lies in the lack of direct correspondence between individual variants from the model population and individual biological cells from the pool of cardiomyocytes measured. This leads to model populations that do not align with biomarker distributions which they have not been calibrated to fit beforehand and undermines their predictive capacity. By contrast, sample-specific approaches are attractive for their one-to-one correspondence between biological cells and tailored mathematical models. Customized models facilitate quantification of the internal state of specific cells, calculation of characteristics that are either unmeasured or difficult to assess and prediction of behaviour under diverse external conditions. However, developing sample-specific models is resource intensive, requiring ample experimental data to constrain the models and substantial effort for subsequent parameter estimation. Consequently, this approach has been primarily limited to single or very few cells and rarely employed to study inter-cell variability.

### 1.3. Goals

The general goal of this article is to combine the advantages of sample-specific and population-based approaches. This has now become possible owing to increases in computing power and gradual improvement in classical and probabilistic algorithms for parameter estimation [23]. More importantly, this has been enabled by recent advances in optics-based techniques for cardiac electrophysiology [24] that make it possible to develop high-throughput automatic and semi-robotic platforms capable of recording transmembrane voltage traces in several thousand uncoupled cardiomyocytes per hour [25–28]. The particular aim of the study is to develop and apply such a hybrid cell-specific population-based methodology for statistical analysis and interpretation of new experimental recordings of cardiomyocyte AP waveforms from our group. In our recent work [29], voltage-sensitive fluorescent dyes were employed to capture action potential waveforms from nearly 500 cardiomyocytes isolated from the left ventricular wall of 12 rabbit hearts, revealing substantial variability among cells. In particular, the durations of action potentials at 90% repolarization (APD_90_) had wide (40–50 ms) interquartile ranges indicating variability considerably exceeding that of median APD_90_ values across different animal hearts and within the endo-epicardial and apical-basal regions of each heart. A conventional population-based analysis of the experiment of [29] was then performed. The detailed ionic current model of Shannon *et al*. [30] was selected as a baseline mathematical representation of the rabbit myocytes, 50 000 model variants with randomly sampled values of eight sensitive parameters were then generated, and the population was calibrated by rejecting variants falling outside the experimentally measured histogram distribution of APD_90_ values. However, a large amount of valuable experimental information was discarded in the process. Notably, while complete AP traces comprising voltage values recorded at a frequency of 10 kHz (i.e. 10 000 voltage values per second) were available for all cells, only a single value (APD_90_) per cell was used. Model variants were not constrained to reproduce cell-specific APD_90_ values, only to return values consistent with an experimental-like histogram distribution of this biomarker. Consequently, the model population did not reproduce the experimentally measured distributions of other measured biomarkers, e.g. APD_30_ and APD_50_. In the present article, we attempt to improve on this approach by constraining parameter values to find cell-specific model variants that reproduce the entire AP waveforms of individual biological myocytes. We will use a large population of over 1200 myocytes measured for this purpose following optics-based experimental protocols similar to that of [29]. This refined approach ensures a more comprehensive use of the experimental data, enhancing the accuracy and robustness of our analyses and the predictive capacity of both individual models and the overall model population.

## 2. Methodology

To construct an ensemble of cell-specific AP models, we estimate an individualized set of parameter values for each experimentally measured cardiomyocyte. Various inference approaches exist, frequentist as well as Bayesian. Here, we outline our chosen methodology with associated assumptions and notation.

### 2.1. Parameter estimation in dynamical systems

#### 2.1.1. Set-up

To formulate a cell-specific model, we consider each cardiomyocyte as a spatially localized system C described by a set of ordinary differential equations of the form


(2.1) $$\begin{array}{c} \frac{d}{dt}x=f(t,x;\vartheta ),\;x(0)=x_{0},\;y=s(x). \end{array}$$

Here, $t\in {\mathbb{R}}^{1}$ denotes time, $x(t,\vartheta ,x_{0})\in {\mathbb{R}}^{d}$ is a vector of state variables with initial values $x_{0}\in {\mathbb{R}}^{d}$ and $\vartheta \in {\mathbb{R}}^{k}$ is a vector of model parameters, $y(t,\vartheta ,x_{0})\in {\mathbb{R}}^{n}$ is a vector of observable outputs and f and s are functional relationships, often called ‘the model’ in this context. We consider a dataset:


(2.2) $$\begin{array}{c} D:=\{(t_{j},Y_{j}){\}}_{j=1}^{K}, \end{array}$$

of K experimental values $Y_{j}\in {\mathbb{R}}^{n}$ of the observables y measured at discrete times $t_{j}$.

#### 2.1.2. Noise model

Measurements are subject to random observational errors and inherent fluctuations within the complex system C. Thus, we assume that the experimental data values $Y_{j}$ are realizations of normally distributed independent random variables $Y_{j}$ with mean values $E[Y_{j}]=y(t_{j},\theta ,x_{0})$ and unknown but identical variances $\sigma^{2}=\sigma_{j}^{2}$, i.e.:


(2.3) $$\begin{array}{lcr} & Y_{j}∼N(y(t_{j},\theta ,x_{0}),\sigma^{2}). & \end{array}$$

These commonly made assumptions are justified by the universality of the Gaussian probability distribution N. There is no essential distinction between the unknown variance parameter $\sigma^{2}$, the initial values $x_{0}$ and the model parameters ϑ and we group them in the vector $\theta \subseteq (\vartheta ,x_{0},\sigma )\in {\mathbb{R}}^{l}$ with l≤d+k+1. We now turn to their estimation.

#### 2.1.3. Maximum likelihood

We invoke the maximum likelihood principle to: (i) find point estimates $\hat{\theta }$ of these parameters, (ii) measure the standard errors $\sigma_{\hat{\theta }}$ of the estimates, and (iii) quantify the goodness of fit. Under our assumptions, the likelihood function of the data D, defined as the joint probability density P to measure $Y=\left\{Y_{j},j=1\ldots K\right\}$ considered as a function of the parameters θ, is


(2.4) $$\begin{array}{lrlr} & L(Y;\theta )= & P(Y|\theta )=\prod_{j=1}^{K}P(Y_{j}|\theta )=\prod_{j=1}^{K}\frac{1}{\sqrt{2\pi \sigma^{2}}}\exp\left(-\frac{1}{2}\frac{(Y_{j}-y(t_{j},\vartheta ,x_{0}){)}^{2}}{\sigma^{2}}\right). & \end{array}$$

#### 2.1.4. Point estimates

Proceeding from the definition of likelihood, the maximum-likelihood principle postulates that the best point estimate $\hat{\theta }$ of the parameter values is given by the values of θ for which L(Y;θ) attains its global maximum:


(2.5) $$\begin{array}{lcr} & \hat{\theta }=\arg\;\max_{\theta \in \Theta }\;L(Y;\theta ), & \end{array}$$

where $\Theta \subseteq {\mathbb{R}}^{l}$ is an appropriately constrained region of the parameter space. The evaluation of the maximum-likelihood estimator (2.5) now becomes a mathematical optimization problem that may be solved by numerous methods [31].

#### 2.1.5. Errors of estimation

We assume that errors in numerical optimization are negligible in comparison to errors in the estimation of θ that arise from experimental noise. Thus, we seek the standard error of the estimates as the square root of the variance $\text{Var}[\hat{\theta }(Y)]$. This, in turn, can be related to the variance ${\hat{\sigma }}^{2}$ of the voltage measurements, which can equivalently be interpreted as the standard error of voltage estimation, and found as a component of equation (2.5). Approximating the data Y by the model y and then the relationship $\hat{\theta }(y)$ by the linear terms of its Taylor expansion about the expectation E[y], we find that standard errors of the estimates can be measured by


(2.6) $$\begin{array}{lrr} & \sigma_{\hat{\theta }}=\hat{\sigma }\sqrt{\text{diag}([J^{T}J{]}^{-1})},\;\;\;\;\;J=\nabla_{\theta }y{|}_{\hat{\theta }}, & \end{array}$$

where $\nabla_{\theta }$ denotes the gradient of partial derivatives with respect to θ and T denotes a matrix transpose. Here, we have taken advantage of the inverse function theorem and expressed the parameter covariance matrix in terms of the Jacobian matrix J of the inverse relation y(θ) as this is more easily evaluated by a minor extension of problem (2.1).

#### 2.1.6. Goodness-of-fit

To assess the goodness-of-fit, we exploit the fact that if $Y_{j}$ are normally distributed as assumed, and if $y(t_{j},\theta )$ is linear in θ, then the sampling distribution of the sum of squared errors


(2.7) $$\begin{array}{lcr} & {\hat{\chi }}^{2}(\hat{\theta })=\sum_{j=1}^{K}\frac{(Y_{j}-y(t_{j},\hat{\vartheta },{\hat{x}}_{0}){)}^{2}}{{\hat{\sigma }}^{2}}, & \end{array}$$

must be a χ^2^ distribution with ν=K−l degrees of freedom, see [32]. Specifically, let $H_{0}$ be the null hypothesis asserting assumptions are correct, and the quality of fit is good. To test $H_{0}$, we calculate the probability p under $H_{0}$ of obtaining a value $\chi_{\nu }^{2}$ that is larger than the value ${\hat{\chi }}^{2}(\hat{\theta })$ measured at the best parameter estimates $\hat{\theta }$:


(2.8) $$\begin{array}{lcr} & p=\text{Pr}(\chi_{\nu }^{2}\geq {\hat{\chi }}^{2}(\hat{\theta })\;|\;H_{0})=\int_{{\hat{\chi }}^{2}(\hat{\theta })}^{\infty }P(\chi_{\nu }^{2})\;d\chi_{\nu }^{2}, & \end{array}$$

where $P(\chi_{\nu }^{2})$ is the probability density function of the χ^2^ distribution with ν degrees of freedom. We reject $H_{0}$ at significance level γ if p<γ. Linearity of y(t;θ) is not satisfied by equation (2.1), but classical texts [33] advise that this test is also acceptable in non-linear cases.

#### 2.1.7. A population of isolated cells

Finally, to extend the analysis to population level, we consider a set of N cardiomyocytes with associated experimental data:


(2.9) $$\begin{array}{c} C=\{C_{i}{\}}_{i=1}^{N},\;\;D=\{D_{i}{\}}_{i=1}^{N}. \end{array}$$

We find parameter estimates (equation (2.5)), their standard errors (equation (2.6)), and evaluate the test (equation (2.8)) for each one. The population of system-specific models consists of all accepted fits:


(2.10) $$\begin{array}{c} M=\{{\hat{\theta }}_{i}\pm \sigma_{\hat{\theta },i}{\}}_{i=1}^{N},\;\;\text{s.t.}\;\;\;p>\gamma . \end{array}$$

### 2.2. Fluorescence recordings of action potential waveforms

We fitted newly recorded AP waveforms from 1228 rabbit ventricular myocytes. Cardiomyocytes were obtained from eight male New Zealand white rabbits. Enzymatic cardiomyocyte isolation and fluorescence-based recording of transmembrane voltage from individual cardiomyocytes were performed as previously described in [29]. Myocytes isolated from the free wall of the left ventricle were loaded (16 min, room temperature) with 0.08 μl ml^−1^ FluoVolt (Thermo Fisher Scientific). For recordings, myocytes were bathed in Krebs-Henseleit solution containing (in mM): 120 NaCl, 1.8 CaCl_2_, 20 HEPES, 5.4 KCl, 0.52 NaH_2_PO_4_, 3.5 MgCl_2_⋅6H_2_O, 20 taurine, 10 creatine and 11 glucose (pH 7.4 at 37°C). Myocytes were subjected to field stimulation with 40 V and 2 ms pulses at a frequency of 2 Hz. Cells were stimulated for 5 min before recordings. Fluorescence intensity was then measured at 10 kHz frequency for a further 2.5 s, yielding a train of five AP. These were then temporally averaged to provide a single waveform for each cell. The beat-to-beat variability was monitored and assessed as described previously in [29] and was less than 2% for measurements from individual myocytes. All recordings were made at 37°C. Cells can be split into sub-populations from distinct apical/basal and endo/mid/epicardial sub-regions and from different animals. However, here we regard all measured cells as a single large myocyte population C with associated experimental data D, consisting of individual time series $D_{i}$ of fluorescence intensities $V_{i,j}$ measured at times $t_{j}$:


(2.11) $$\begin{array}{c} D={\left\{D_{i}=\{(t_{j}=j\Delta t,V_{i,j}),\;\Delta t=10^{-4}\text{s}{\}}_{j=1}^{K}\right\}}_{i=1}^{N},\;K=5000,\;\;N=1228. \end{array}$$

**Figure 1.**
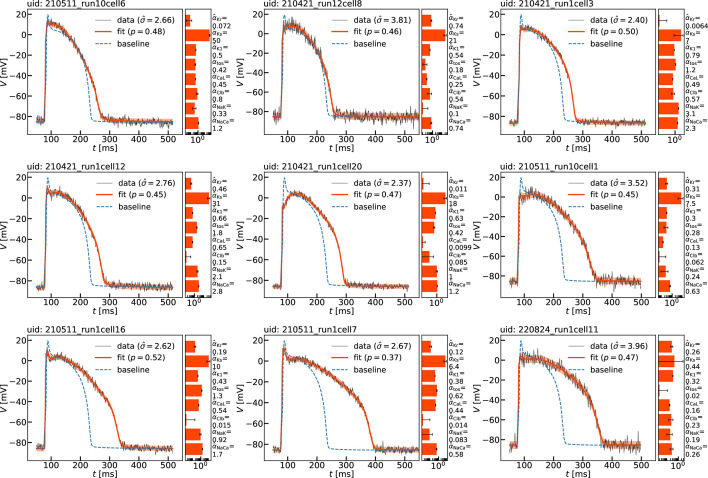
Examples of parameter estimations for nine typical biological myocytes. Cells are identified by unique identifiers (uid). The experimentally measured action potential waveforms $\left\{(t_{j},V_{j}),j=1,\cdots \;,5000\right\}$ are shown by thin black lines. AP waveforms $\hat{V}(t)$ computed from the accepted fits of the Shannon model (equation (2.12)) are shown by thick orange-red lines. Estimates of the standard deviation $\hat{\sigma }$ of noise in voltage measurements are quoted in the legends and are illustrated by the width of the semi-transparent orange-red strips centred on $\hat{V}(t)$. The corresponding point estimates $\hat{\alpha }$ of the parameter values and their standard errors $\sigma_{\hat{\alpha }}$ found by equations (2.5) and (2.6), respectively, are quoted and illustrated in a separate bar chart for each cell. The goodness-of-fit values p measured by equation (2.8) are listed in the legends. The action potential waveform $\overset{ˇ}{V}(t)$ of the baseline Shannon model is shown by blue dashed lines for comparison.

Examples of nine single averaged AP waveforms are shown in figure 1 and those of all accepted biological cells are included in the electronic supplementary material, figure S1.

### 2.3. Baseline action potential model

#### 2.3.1. Model description

The Shannon *et al*. [30] model of the rabbit ventricular myocyte is used as a baseline model for the experimental data D. In this model, cells are represented as a collection of four compartments: sarcoplasmic reticulum, junctional cleft, subsarcolemmal space and cytosolic bulk. Ions of species Ca^2+^, Na^+^, K^+^, and Cl^−^ are exchanged between compartments and with the extracellular space by facilitated diffusion and active transport. The model characterizes the instantaneous state of a cell by a set of 38 state variables including ion channel gating variables, ryanodine receptor variables and concentrations of each of the four ionic species in free and bound states and for each of the four compartments. Here, these variables are denoted by a vector $z\in {\mathbb{R}}^{38}$ and obey a set of nonlinear ordinary differential equations with rates given by a vector field g. The ion transport fluxes give rise to 15 electric currents, namely fast Na^+^ current $I_{\text{Na }}$, L-type Ca^2+^ current $I_{\text{CaL }}$, rapid and slow components of the delayed rectifier K${,}^{+}$ current $I_{\text{Kr }}$ and $I_{\text{Ks }}$, respectively, inward rectifier K^+^ current $I_{\text{K1 }}$, fast and slow transient outward K^+^ currents $I_{\text{tof }}$ and $I_{\text{tos }}$, respectively, Ca^2+^ -activated Cl^−^ current $I_{\text{ClCa}}$, Na^+^/Ca^2+^ exchanger current $I_{\text{NaCa }}$, Na^+^/K^+^ pump current $I_{\text{NaK }}$, sarcolemmal Ca^2+^ pump current $I_{\text{Cap }}$ and background Na^+^, K^+^, Ca^2+^, and Cl^−^ currents $I_{\text{Nab }}$, $I_{\text{Kp }}$, $I_{\text{Cab }}$, $I_{\text{Clb }}$, respectively, denoted by $I_{k},k=1\ldots 15$ below. The ion currents are simultaneously modulated by and drive changes in the voltage V across the sarcolemma which, in turn, is modelled as a circuit with a capacitor and a resistor connected in parallel. The experimental cells are excited by an additional stimulus current, $I_{\text{stim}}$, in the form of a train of rectangular pulses. With these notations, the model of Shannon *et al*. [30] can be specified by setting in equation (2.1):


(2.12) $$\begin{array}{lrr} & x=[V,z{]}^{T},y=s(x):=V,f=[\sum_{k=1}^{15}I_{k}(V,z;\vartheta )+I_{\text{stim}}(t,\vartheta ),g(V,z;\vartheta ){]}^{T}, & \end{array}$$

where $\vartheta \in {\mathbb{R}}^{140}$ is a vector of 140 model parameters including the stimulus duration, amplitude and frequency. Initial values of all state variables must be included, extending the vector of parameters to 179, i.e. $[\vartheta ,x_{0}{]}^{T}\in {\mathbb{R}}^{179}$. Specific parameter values and algebraic expressions for the currents and the nonlinear functions f and g are provided in the original publication [30], and we use an error-free machine readable implementation from the CellML model repository [34]. The only modification made is that the reversal potential of sodium ions across the sarcolemma is changed from a Nernst equation to the fixed value $E_{\text{Na,SL}}=-15\text{mV}$ to mimic experimental waveforms $D_{i}$ where the ‘spikes’ of the peak voltages are systematically observed to be smaller than those of the original Shannon model. This implementation reflects the uncertainty as to the electrical conditions that initiate the AP and the model’s ability to reproduce aspects of field stimulation and explains why this study is focused on fitting the secondary repolarization phase.

#### 2.3.2. Numerical solution

The model solution $x(t;\vartheta ,x_{0})$ and observables $y(t,\theta ,x_{0})$ are required to evaluate the likelihood function (2.4) at given parameter values. Solutions are obtained numerically using the CVODES method from the SUNDIALS suite of nonlinear and differential equation solvers [35] with absolute and relative tolerance settings of $10^{-6}$ and $10^{-8}$, respectively. The Myokit ‘interface to cardiac cellular electrophysiology’ [36] is used to access CVODES.

### 2.4. Estimands and optimization details

#### 2.4.1. Estimands

It is computationally infeasible to include all 179 parameters of the Shannon model in the parameter estimation problem (equation (2.5)). In this study, we keep the vast number of parameters fixed to their original values and seek to calibrate only the following eight model parameters and the standard variation of noise:


(2.13) θ=[GKr,GKs,GK1,Gtos,GCaL,GClb,INaK,INaCa,σ]T∈ℝ9.

These eight parameters were chosen following our earlier local sensitivity analysis of this model published in [29]. Here, subscripts denote ion currents as introduced in §2.3, with G being the maximal conductance and I being the maximal density of currents with Ohmic and with Goldman-Hodgkin-Katz mathematical formulations, respectively. In the following, results are quoted as a proportion $\alpha \in {\mathbb{R}}^{9}$ of the published baseline values $\overset{ˇ}{\theta }$ and the estimates of the actual cell-specific parameter values can be obtained by taking the Hadamard element-wise product $\theta =\alpha ⊙\overset{ˇ}{\theta }.$ The proportion factors $\alpha_{k}$ represent ‘relative strengths’ of currents compared to the baseline, as used for instance in [37]. The factors $\alpha_{k}$ are assumed positive in order to preserve both model dynamics and the physiological interpretation of the calibrated parameters.

#### 2.4.2. Optimization details

The global maximization (equation (2.5)) of the logarithm of the likelihood function (equation (2.4)) is performed using the covariance matrix adaptation evolution strategy algorithm (CMA-ES) of [38] as implemented in the Python module PINTS [39]. The CMA-ES is a gradient-free method designed for high-dimensional, ill-conditioned and non-convex problems. It initiates a population of candidate estimates by sampling from a multivariate normal distribution with a mean and a covariance matrix given by an initial guess. It evaluates the log-likelihood of these candidates by simulating the Shannon model (equation (2.12)) and comparing the computed and the measured voltage traces as per equation (2.4). It then selects a sub-population of the most likely candidates and uses them to compute an updated mean and covariance matrix, thus effectively finding the direction of higher likelihood. The steps of sample generation, selection and update are then repeated until given convergence criteria are met. A population of 96 initial candidates is used with means centred at unity corresponding to the published Shannon model baseline values and with diagonal covariance matrices with variances set to 16.66. The variance values represent one-sixth of the boundary ranges for the parameter search which were taken as $10^{-4}$ to $10^{2}$ for all parameters. The optimization is terminated when estimates exhibit a relative change of less than $10^{-6}$ over the last 100 iterations of the algorithm.

## 3. Results and discussion

We now proceed to describe the population of cell-specific action potential models obtained by fitting the Shannon *et al*. [30] model to voltage-sensitive fluorescence measurements in rabbit ventricular myocytes.

### 3.1. Illustrative demonstration

To illustrate the parameter inference procedure on specific examples, we discuss first a small subset of nine rabbit ventricular myocytes. Figure 1 shows visualizations of: (i) the available single-cell experimental data, (ii) the accepted fits with their goodness-of-fit measures, (iii) the inferred model parameter estimates with their standard errors, along with (iv) a direct comparison to the baseline Shannon model for these nine typical myocytes. Full details for the remaining 1180 accepted cells are included in the electronic supplementary material, figure S1 and tables S3 and S4.

#### 3.1.1. Features of experimental waveforms

In common with the APs of all excitable cells, ventricular APs are large transient excursions away from electric potential equilibrium that exists across sarcolemmas [13]. A good example of the generic morphology of the ventricular AP is provided by the voltage component $\overset{ˇ}{V}(t)$ of the solution to the baseline Shannon model plotted in figure 1. The experimentally measured waveforms $V_{j}(t)$ exhibit similar behaviour but show several distinctive features as seen in figure 1. In particular, we note that the experimental APs $V_{j}(t)$ plotted in the figure are longer than that of the baseline model $\overset{ˇ}{V}(t)$ as measured by their AP duration (APD_90_). This is also true for the majority of all 1180 biological cells. The duration APD_90_ is defined as the time interval between depolarization upstroke and repolarization downstoke measured at 90% of the waveform amplitude; durations APD_*x*_ can be defined similarly. Another characteristic feature of the measured traces $V_{j}(t)$ is the absence of significant spikes, and rather weak notches afterwards. Some experimental waveforms feature no spikes at all as seen, e.g. in cell uid: 210421_run1cell20. This can be understood as a case of a fast sub-threshold but slow over-threshold response to the excitation stimulus [40], where for the particular cell the stimulus current has insufficient amplitude or duration to trigger a response in the fast sodium current $I_{\text{Na,SL}}$ but is large enough to trigger a response in other slower currents so that the voltage transitions to the quasi-stable manifold of the plateau ‘from below’. It is the characteristic absence of pronounced spikes in experimental waveforms that lead us to reduce the baseline value of the reversal potential $E_{\text{Na,SL}}$ of the sarcolemmal sodium current to −15 mV, as mentioned in §2.3. All other effects of this change on the baseline model are negligible as demonstrated in the electronic supplementary material, figure S2.

#### 3.1.2. Effect of noise

Random noise appears to have different signal-to-noise ratio in different cells as seen in the examples of figure 1. The random noise has a characteristic time scale shorter than the time scales along the slow pieces of the AP trajectory, e.g. slower than the time scale of evolution on the plateau and the resting potential, but comparable to the fast time scale of the upstroke. This makes the noise transversal to the slower pieces of theAP resulting in difficulties in ascertaining exact values of voltage along the plateau and at resting equilibrium without resorting to long temporal running averages. It also makes it difficult to recognize waveform features that are comparable in time scale and amplitude to noise as it is impossible to time-average there. The spikes, and to a lesser extent the notches, are the main such morphological features and, as a result of noise, the peak voltage $V_{\text{peak}}$ cannot be accurately determined. On the other hand, processes on faster time scales and with larger amplitudes than noise, e.g. the upstroke and the APD_90_, are less affected by noise and can be determined relatively accurately.

The electronic supplementary material, figure S3 illustrates further the properties of experimental noise. Sub-plot (a) provides a histogram of residual differences between the true experimental values of the voltage and the model estimated values of the voltage for all moments in time, $\left\{e_{i}=V_{j}-\hat{V}(t_{j}\right)$, i=1,⋯,5000}. The mean value of the histogram sample deviates insignificantly from zero, which is owing to the finite size of the sample (5000). The standard deviation of the sample is identical to the standard deviation (standard error of estimation of voltage) found by optimization. A Gaussian distribution with these parameter values closely captures the shape of the histogram as shown in the figure. This demonstrates that the assumption of normality of errors is well satisfied. Sub-plot (b) shows the autocorrelation (Pearson’s correlation coefficient r) between voltage residual values at moments t and t+lagΔt. Non-negligible correlation is observed with the preceding 10 to 15 values which suggests that the assumption of independence and identical distribution of errors is not well satisfied. A more general autoregressive (integrated) moving-average noise model may have been more accurate to use. Inevitably, this involves estimating a larger number of parameters and will be left for future refinements.

#### 3.1.3. Fluorescence to voltage mapping

Voltage-sensitive fluorescence measurements do not provide absolute voltage values. To convert fluorescence intensity to voltage, we have chosen to map the time-averaged fluorescence intensity at the plateau to 0 mV and the time-averaged fluorescence intensity at rest to −86 mV with linear scaling between. The plateau is chosen as noise prevents accurate capturing of the signal spike as discussed above. The values 0 and −86 mV are the values of ${\overset{ˇ}{V}}_{\text{plateau}}$ and ${\overset{ˇ}{V}}_{\text{rest}}$ of the voltage in the baseline Shannon model, respectively. The examples of figure 1 demonstrate that this works well.

#### 3.1.4. Model fits

The accepted model fits for the nine cells of our illustrative subset are shown in figure 1. The estimated parameter values $\hat{\theta }$ found by maximizing the likelihood function via equation (2.5) are explicitly stated as proportions $\hat{\alpha }$ relative to the baseline values $\overset{ˇ}{\theta }$ and illustrated by bar charts for each cell.

The standard errors of these estimates, denoted as $\sigma_{\hat{\alpha }}$, were determined using equation (2.6) and are depicted as error bars overlaid on the bar-chart values in figure 1. Since numerical errors are assumed negligible, these standard errors are measures of uncertainty in the estimation process. Uncertainty is owing to random noise and to model selection choice. The latter manifests itself here as alternative possibilities for selection of the number of parameters to be estimated within the chosen baseline model [30]. It is pleasing to find that, on average, six out of the eight estimates are obtained with small uncertainty for the cells in the illustrative subset. However, a few parameter estimates consistently exhibit large standard errors across all fits, and this uncertainty varies across the estimands for different cells. This issue is further explored in the subsequent subsection.

The synthetic AP waveforms computed from the Shannon model using the quoted cell-specific parameter estimates are superimposed onto the experimental waveforms in figure 1, displaying a close match across all cells and AP phases. The excellent visual goodness-of-fit is substantiated by evaluating the formal measure (equation (2.8)). Values of p are given in the figure and are close to 0.5 for all nine cells. Recall that p represents the probability of obtaining a weighted sum of squared errors $\chi_{\nu }^{2}$ larger than the value $\chi_{\nu }^{2}(\hat{\theta })$ actually measured in a final fit under the null hypothesis that the assumed model is correct. Since the objective is to minimize $\chi_{\nu }^{2}$, large values of p are considered good fits, see discussion in chapter 15 of [33].

The standard deviation $\hat{\sigma }$ of the experimental signal from the synthetic mean is a measure of experimental noise. This quantity is estimated as a component of the parameter vector during log-likelihood maximization as mentioned above. The estimated values of $\hat{\sigma }$ for the nine cells in our illustrative subset are visualized by semi-transparent strips of width $2\hat{\sigma }$ centred on the model synthetic waveforms in figure 1. When superimposed onto the noisy experimental traces, these values and the corresponding strips they define appear to capture noise levels very well.

Finally, we mention that maximum likelihood estimation takes approximately 30 min of wall clock time when running a parallel implementation of the CMA-ES method with 96 threads on a small multi-user computing server. Exact computation times depend on the data being fitted, proximity of the initial guess to maximum likelihood, the random nature of the CMA-ES method itself, but typically convergence is achieved in 300 to 500 generations of the CMA-ES method of which 100 have absolute change smaller than $10^{-6}$ as required by the imposed termination criterion.

#### 3.1.5. Model analysis and prediction

**Figure 2.**
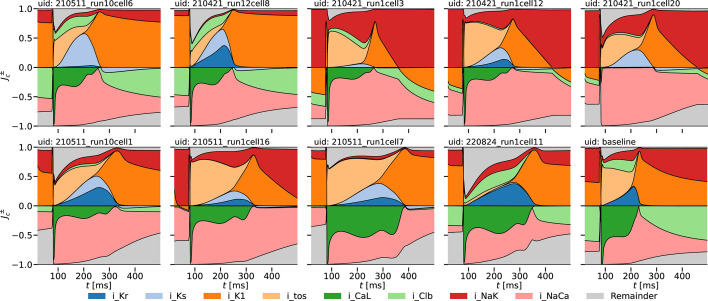
Normalized positive and negative cumulative currents $J_{c}^{\pm }$ as functions of time computed from the cell-specific models of the nine myocytes shown in figure 1. The cumulative currents are defined by equation (3.1*a*) and coloured as specified in the figure legend. The cumulative currents of the Shannon baseline model are also shown for comparison.


The chief purpose of a mathematical model is to conceptualize a real-world system and to enable its formal analysis and forward prediction. To illustrate this trivial remark, we plot in figure 2 normalized positive and negative cumulative currents $J_{c}^{\pm }$ for the nine cells illustrated in figure 1. These quantities are defined as

(3.1*a*) $$\begin{array}{r} {\left\{J_{c}^{\pm }(t)=\pm \sum_{k=1}^{c}I_{k}^{\pm }(t)/\sum_{k=1}^{K=15}I_{k}^{\pm }(t)\right\}}_{c=1}^{8},\;\;I_{k}^{+}(t)=\max(I_{k}(t),0),\;\;\;I_{k}^{-}(t)=\min(I_{k}(t),0), \end{array}$$

where $I_{k}$ are the Shannon *et al.* [30] model currents indexed by the elements of the ordered set:


(3.1*b*) $$\begin{array}{lcr} & [k{]}_{1}^{15}=[\text{Kr, Ks, K1, tos, CaL, Clb, NaK, NaCa, Na, Nab, tof, ClCa, Cab, Cap, Kp}]. & \end{array}$$

The cumulative currents preserve their strict ordering for all time, i.e. $|J_{c}^{\pm }(t)|<|J_{c+1}^{\pm }(t)|$, c=1⋯7. When cumulative currents are plotted in reverse order from the largest in the background to the smallest in the foreground and as functions of time, complement regions between the curves of $J_{c}^{\pm }(t)$ and $J_{c+1}^{\pm }(t)$ represent the contribution of Shannon current $I_{c+1}(t)$ added to the contribution of the preceding c currents. Thus, the cumulative currents are an equivalent representation of the physiological ionic currents in the model with the advantage that they can be conveniently plotted one over another, the positive and negative parts of currents can be plotted separately and the relative contribution of each current to the total can be shown. For comparison, the cumulative currents corresponding to the baseline Shannon model are shown in a ‘mirror image’ against each myocyte to illustrate the predicted differences in internal ion dynamics of each cell. We point to the fact that the cumulative contribution of those currents that have been kept fixed during parameter estimation is small. This cumulative ‘remainder’ current is plotted in grey in figure 2. The actual ionic currents in the selection of models are shown in the electronic supplementary material, figure S4. We note in figure 2 that the background chloride current near the resting state can be as low as 0% of the total for some cells or as high as 90% of the total for others. However, the electronic supplementary material, figure S4 shows that the total current at rest is very nearly zero. Since ionic currents have not been measured in our experiments, this calculation presents an example of model forward prediction. Similarly to the example of figure 2, cell-specific Shannon models can be used, with equal ease, to derive and compute additional quantities that are impossible to, or have not been, experimentally measured. For instance, time series of ionic concentrations can be easily calculated, and cellular responses under different conditions including drug treatments can be readily predicted. Detailed applications of such type will be considered in future work.

The features and the overall quality of parameter estimation, illustrated here on the nine example myocytes, are typical for the entire population of 1228 fitted cells, as evidenced in the electronic supplementary material.

### 3.2. Synthetic data test

**Figure 3.**
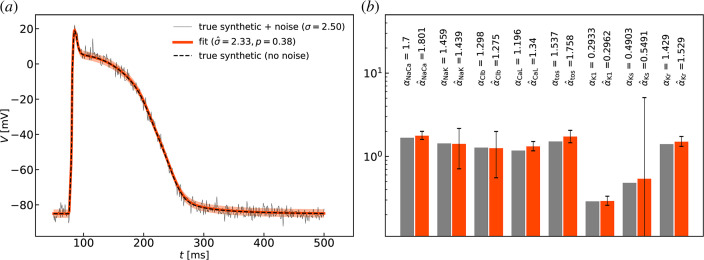
Fitting to synthetic data. Given randomly selected, but known, parameter values (grey barchart in panel (*b*)) a synthetic action potential trace was generated (black dashed curve in panel (*a*)). Gaussian random noise with mean 0 and standard deviation σ=2.5 was added to values of $V_{i}$ to produce a synthetic noisy action potential trace [thin black curve in (*a*)). The noisy signal was re-fitted as described to produce a fitted AP trace [thick red curve in (*a*)). The red barchart in (*b*) shows the point estimates of the parameter values with standard errors shown in black error bars with end caps.

To further validate the inference methodology, we performed parameter fitting of synthetic data as illustrated in figure 3. The test allows evaluation of absolute errors which are not available when fitting experimental data.

#### 3.2.1. Synthetic data generation

We drew random values for each of the eight parameter estimands $\alpha_{k}$ of equation (2.13) from a continuous uniform distribution U([0.1,2]). The specific values are given in figure 3*b*. With these, we computed a numerical solution of the Shannon model (2.12) to generate a ‘synthetic’ clean AP waveform V(t). We added to this signal normally distributed random noise with standard deviation σ=2.5 to obtain a synthetic noisy AP V(t)+v(t), where $v(t)∼N(0,\sigma^{2})$. There was no specific consideration in choosing the interval of the uniform distribution to be [0.1,2] other than to avoid ‘non-action potential’ solutions which often occur when parameter values are taken far from the baseline values of unity. The value of the standard deviation was selected to be similar to the ones found in the illustrative examples shown in figure 1. The resulting synthetic AP trace without and with noise is plotted in figure 3 and the noisy one is visually indistinguishable from typical measurements as seen in figure 1 and in theelectronic supplementary material, figure S1.

#### 3.2.2. Quality of fit

The synthetic noisy AP was then re-fitted following steps identical to those involved in the parameter estimation for biological myocytes. The results of the test are presented in figure 3. We find absolute error in estimating the standard deviation of noise to be $|\sigma -\hat{\sigma }|=0.17$ corresponding to a relative error $|\sigma -\hat{\sigma }|/\sigma =0.068$. We find similarly small relative errors of the order $O(10^{-2})$ for the values of all estimands as illustrated in figure 3*b* where the estimated parameter values and the absolute errors between estimates and true values of the estimands $|\alpha -\hat{\alpha }|$ are given. These absolute errors are well within, in fact significantly smaller than, the standard errors of estimation determined by equation (2.6). It must be noted that, by contrast, the standard errors of estimation $\sigma_{{\hat{\alpha }}_{k}}$ are particularly large for some estimates. In this instance, ${\hat{\alpha }}_{\text{Ks}}$, and to a lesser degree ${\hat{\alpha }}_{\text{Clb}}$ and ${\hat{\alpha }}_{\text{NaK}}$, have large standard errors even though their values are very accurate estimates of the true ones. We interpret this as an indication that the model solution is not very sensitive to the values of these specific parameters. Naturally, sensitivity varies across the eight-dimensional parameter space and the model solution may be more or less sensitive or insensitive to different estimands. This can be observed in myocytes uid: 210421_run1cell3 and uid: 220824_run1cell11 of figure 1 where ${\hat{\alpha }}_{\text{Kr}}$ and ${\hat{\alpha }}_{\text{tos}}$ show large standard errors of estimation indicating that the model is less-sensitive to these quantities in these instances. However, the small relative errors $|(\alpha_{k}-{\hat{\alpha }}_{k})/\alpha_{k}|$ found in the synthetic test give confidence that estimates are accurate even when parameter uncertainly as measured by $\sigma_{{\hat{\alpha }}_{k}}$ is significant.

The most important test of the quality of the fit is, of course, the agreement between the synthetic clean AP trace V(t) and the trace $\hat{V}(t)$ computed from the re-fitted model using the estimated parameter values. The two traces visually overlap as seen in figure 3*a*. To quantify the difference between them precisely, we computed the values of the absolute and the relative root mean square averages of the errors between the two:


$${\bar{e}}_{V}=1.0\times 10^{-2}\text{mV},\;\;\;\;{\bar{e}}_{V}/V_{\text{amp}}=1.0\times 10^{-4},\;\;\;(V_{\text{amp}}=103.78\text{mV}).$$

These are defined as usual by


$${\bar{e}}_{V}=\frac{1}{N}\sum_{i=1}^{N=5000}\sqrt{(V(t_{j})-\hat{V}(t_{j}){)}^{2}},\;\;\;\;\;V_{\text{amp}}=\max_{t}V(t)-\min_{t}V(t).$$

In summary, the small relative errors from the known true values in this synthetic test demonstrate the excellent quality of the fit shown in figure 3. While true errors are unknown for biological cells, we believe that fits are of a similarly good quality for all cells.

### 3.3. Bayesian inference test

To further test the ‘frequentist’ methodology of §2.1, we undertook Bayesian inference for the cells included in figure 1. The Bayesian method provides more accurate values for the standard errors of estimation and insights into parameter uniqueness and identifiability, as well. An example of a similar application of both maximum-likelihood and Bayesian methodologies to canine AP models is presented in [41].

#### 3.3.1. Bayesian inference

The Bayesian approach assumes that, instead of being deterministic, the estimands are random variables with probability distributions given by Bayes’ Theorem:


(3.2) $$\begin{array}{lcr} & P(\theta |Y)=P(Y|\theta )P(\theta )/P(Y). & \end{array}$$

Here, a ‘prior’ probability distribution on the parameters P(θ) is assumed from pre-experiment considerations, then modified by the likelihood function P(Y|θ) of equation (2.4) and normalized by the available experimental ‘evidence’ distribution P(Y) to obtain the ‘posterior’ probability distribution P(θ|Y). The latter fully describes the estimands and can be used to evaluate any requited moments. In particular, the mode and the standard deviation of the posterior can be compared to the frequentist ‘best’ estimate (equation (2.5)) and standard error of estimation (equation (2.6)), respectively.

#### 3.3.2. Sampling

The posterior is rarely available in closed form, but it can be sampled numerically. We used the Haario-Bardenet adaptive covariance Markov chain Monte Carlo method [42] as implemented in [39]; this is an algorithm for sampling involved high-dimensional distributions. For each cell, five Markov chains were initialized at the best values found by optimization with addition of a small amount of independent zero-mean Gaussian noise. Chains were run for 10 000 steps converging to an average scale reduction factor $\hat{R}$ of 1.08 and producing 50 000 samples for each parameter as illustrated in the electronic supplementary material, figure S5.

#### 3.3.3. Bayesian sampling test results

**Figure 4.**
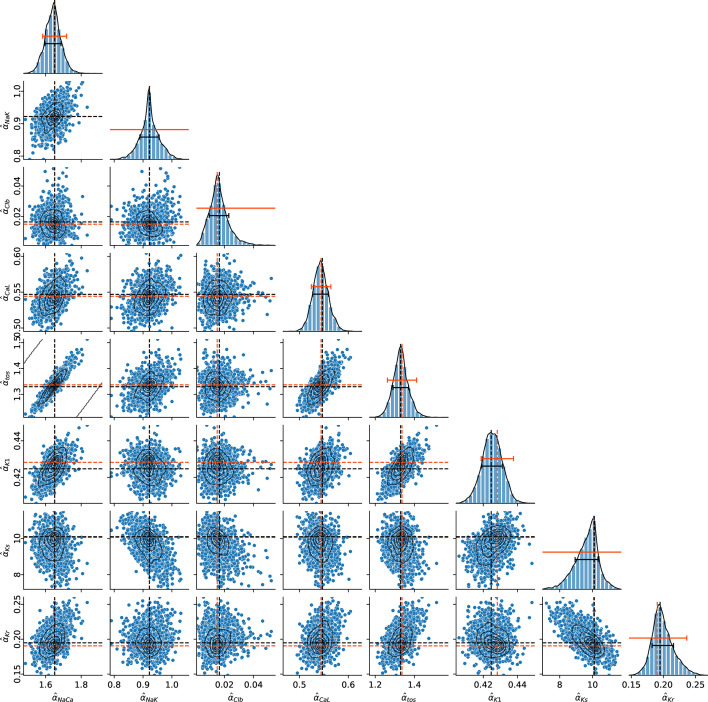
Univariate (on the main diagonal) and bivariate pairwise (lower diagonal part) marginal posterior distributions in the estimand space of cell uid: 210511_run1cell16. Black and orange-red dashed lines show the Bayesian sampling maximum a posteriori probability estimates ${\hat{\alpha }}^{\text{bs}}$, and the global likelihood maximization estimates ${\hat{\alpha }}^{\text{lm}}$, respectively. In the diagonal plots, the black error bars show the standard deviation from mean of the posterior distributions, and the orange-red error bars show the standard errors determined by (2.6). Kernel density estimation contours are shown throughout.

Figure 4 illustrates the posterior probability distribution of the estimated parameters for cell uid: 210511_run1cell16 in one and two-dimensions. The maximum a posteriori probability estimates ${\hat{\alpha }}_{k}^{\text{bs}}$ of Bayesian sampling, and the best estimates ${\hat{\alpha }}_{k}^{\text{lm}}$ of global likelihood maximization agree closely for all estimands, with the largest relative difference being $\tilde{\Delta }{\hat{\alpha }}_{\text{Clb}}=({\hat{\alpha }}_{\text{Clb}}^{\text{bs}}-{\hat{\alpha }}_{\text{Clb}}^{\text{lm}})/{\hat{\alpha }}_{\text{Clb}}^{\text{lm}}=3.2\times 10^{-2}$. The standard deviations from the means of the posterior distributions are smaller, often significantly smaller, than the standard errors of estimation determined from equation (2.6) as illustrated by their comparison in the diagonal plots in figure 4. Thus, the actual uncertainty in parameter estimation is smaller than the rather conservative errors of estimation ${\hat{\sigma }}_{k}$ that we report throughout. This is owing to the classical formula (2.6) being an asymptotic approximation strictly valid only in the limit of infinitely large datasets $V_{j}$. The overestimation of standard errors was also noted in the synthetic data test of §3.2. The pairwise marginal distributions of the majority of estimands show insignificant correlations which is a strong indication that the estimates are indeed unique. The estimands $\alpha_{\text{tos}}$ and $\alpha_{\text{NaCa}}$ are somewhat correlated with their marginal distribution taking the form of a ridge and parameter values along the ridge being nearly equally likely. However, we note that the Bayesian sampling ${\hat{\alpha }}_{k}^{\text{bs}}$ and the likelihood maximization ${\hat{\alpha }}_{k}^{\text{lm}}$ estimates continue to agree well in this case too. The Bayesian sampling results for the rest of the cells from figure 1 are very similar as shown in the electronic supplementary material, table S1.

In summary, Bayesian sampling confirms uniqueness of estimation, indicates a smaller parameter uncertainty than that measured by equation (2.6) and shows excellent agreement with likelihood maximization estimates. Because of this, and the fact that sampling is significantly more expensive computationally, we did not perform Bayesian analysis for the rest of the myocytes.

### 3.4. Cell-specific model population as a random sample of a ‘healthy myocyte’ phenotype

The AP waveforms recorded from 1228 rabbit ventricular myocytes were fitted. Out of these, 1180 models with goodness-of-fit p>0.3 were accepted. The value γ=0.3 is selected by comparison with the goodness-of-fit values of the fits shown in figure 1 which we consider to be good. The electronic supplementary material, figure S1 illustrates the fits for all accepted fits in a format identical to that of figure 1. The rejected fits were approximately 4% of the population size. The experimental AP traces of the rejected fits featured more pronounced versions of the waveforms of cell uid: 220824_run1cell6 and uid: 220824_run1cell20 from the electronic supplementary material, figure S1 and were not captured well by the fitting procedure. We now characterize the population M of parameter estimates as a whole. The elements ${\hat{\alpha }}_{k}$ of M are eight dimensional and consequently visualization and interpretation is challenging.

#### 3.4.1. Population of models as a random sample of a random variable

We consider myocytes $C_{i}$ as elements of a sample space Σ of ‘normal healthy’ cells, and we consider their corresponding Shannon model parameters $\hat{\theta }$ as elements of a measurable space Θ. Then, the set of parameter estimates M represents a random sample from the probability distribution P(θ) of the random variable Σ:F↦Θ, while the parameter estimation process plays the role of the map F.

#### 3.4.2. The cell-specific model population

**Figure 5.**
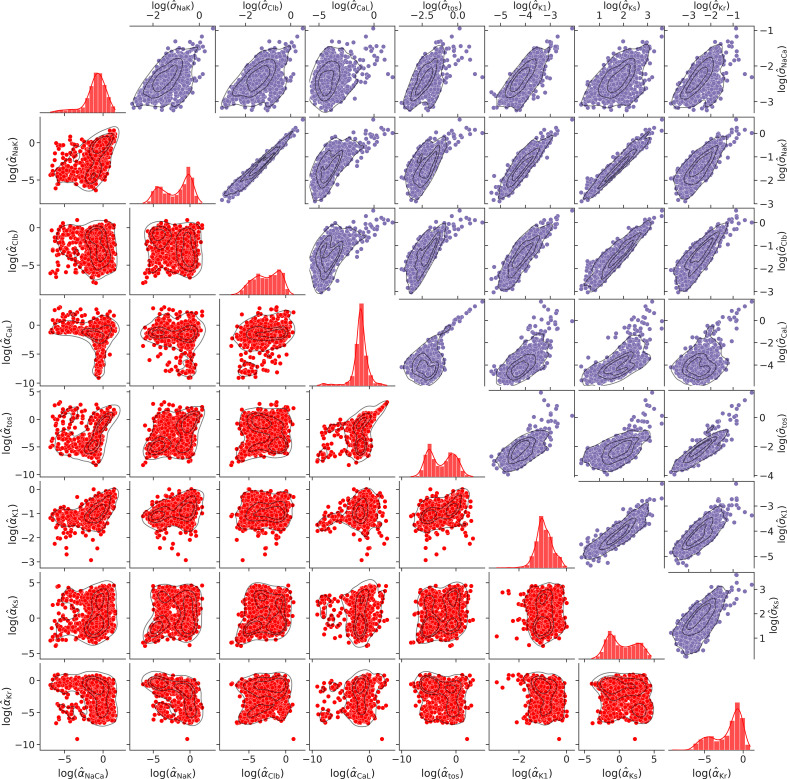
Marginal probability distributions of parameter estimands ${\hat{\alpha }}_{k}$ and their estimation errors ${\hat{\sigma }}_{k}$. Pairwise scatter plots of the estimates ${\hat{\alpha }}_{k}$ and of their standard errors ${\hat{\sigma }}_{k}$ are plotted below and above the grid diagonal in red and in magenta, respectively, for all 1180 accepted myocyte fits. Single-parameter histograms are shown on the grid diagonal in red. Associated marginal kernel density estimations are included throughout. Logarithmic scales are used.

**Figure 6.**
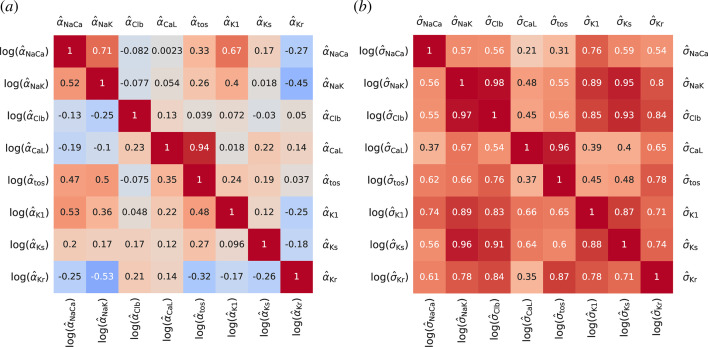
Pairwise sample correlation coefficients r for (*a*) the parameter estimands ${\hat{\alpha }}_{k}$ and (*b*) the standard errors in their estimation ${\hat{\sigma }}_{k}$. For convenience, coefficients of logarithmically transformed and non-transformed samples are plotted in the lower triangular and the upper triangular part, respectively, in both panels.

Attempts to characterize the probability distribution P(θ) of this random variable with the help of the sample M are presented in table 1 and figures 5 and 6 in ‘zero’, one and two dimensions, respectively. Basic summary statistics including ranges, mean, standard deviations and quartiles are listed in table 1 for each of the eight parameter estimands. We find that estimates have large ranges of variation with ${\hat{\alpha }}_{\text{Ks}}$ exhibiting a range of four orders of magnitude. This is in stark contrast to the narrow ranges of variation from the baseline values typically assumed in the literature, e.g. [29]. We use logarithmic scales in the subsequent figures to capture these wide ranges visually and provide a balanced view of the data.

**Table 1.** Sample statistics of the population of parameter estimates M. (Percentages in the column headings denote quartiles of the data. Scientific ‘e’–notation is used for the values of real numbers.)

	count	mean	std	min	25%	50%	75%	max	skewness	kurtosis
${\hat{\alpha }}_{\text{NaCa}}$	1180	7.04e − 1	7.41e − 1	1.09e − 3	2.18e − 1	4.60e − 1	9.16e − 1	4.79e + 0	2.07e + 0	5.11e + 0
${\hat{\alpha }}_{\text{NaK }}$	1180	5.36e − 1	6.48e − 1	1.67e − 3	2.27e − 2	2.97e − 1	8.44e − 1	5.22e + 0	1.98e + 0	6.72e + 0
${\hat{\alpha }}_{\text{Clb }}$	1180	2.24e − 1	3.02e − 1	6.22e − 4	1.97e − 2	8.45e − 2	3.42e − 1	2.83e + 0	2.42e + 0	9.90e + 0
${\hat{\alpha }}_{\text{CaL }}$	1180	4.98e − 1	1.40e + 0	1.11e − 4	1.42e − 1	2.22e − 1	3.68e − 1	2.21e + 1	9.15e + 0	1.04e + 2
${\hat{\alpha }}_{\text{tos }}$	1180	5.53e − 1	1.43e + 0	2.51e − 4	7.80e − 3	6.97e − 2	5.41e − 1	2.30e + 1	7.91e + 0	8.90e + 1
${\hat{\alpha }}_{\text{K1 }}$	1180	4.17e − 1	1.47e − 1	5.33e − 2	3.19e − 1	3.82e − 1	4.86e − 1	1.01e + 0	1.01e + 0	1.11e + 0
${\hat{\alpha }}_{\text{Ks }}$	1180	8.45e + 0	1.53e + 1	1.94e − 2	2.79e − 1	1.08e + 0	9.84e + 0	1.00e + 2	2.82e + 0	9.28e + 0
${\hat{\alpha }}_{\text{Kr }}$	1180	3.68e − 1	3.91e − 1	1.07e − 4	2.87e − 2	2.68e − 1	5.73e − 1	2.54e + 0	1.56e + 0	3.46e + 0

The central tendency, dispersion and range of values provided in table 1 are far from sufficient to capture the complexity of the dataset M. Univariate marginal distributions of all Shannon parameter estimands are shown in the diagonal panels of figure 5 in the form of histograms and Gaussian kernel density estimations. With the exception of ${\hat{\alpha }}_{\text{K1}}$, ${\hat{\alpha }}_{\text{CaL}}$ and ${\hat{\alpha }}_{\text{NaCa}}$, which have long tails of outliers towards small values, the distributions of all estimands exhibit pronounced bimodality. Skewness and kurtosis values are also provided in table 1.

To reveal inter-variable correlations and clustering, bivariate joint marginal distributions are visualized in figure 5 in the form of scatter plot histograms and with contours of associated two-dimensional Gaussian kernel density estimations. These are arranged in a grid of panels where estimates for each Shannon parameter estimand are plotted on the y-axes across one of the rows of the grid as well as on the x-axes across one of its columns. This grid arrangement is commonly known as a pairplot or correlogram and represents a comprehensive two-dimensional view of the dataset M. The uni and bivariate joint distributions shown in figure 5 are marginal as all other estimands vary simultaneously with the one, or the pair, that is being plotted. There are only weak, if any, linear correlations between the parameter estimands. This is further quantified in figure 6*a* by a map of pairwise correlation coefficients. We recall that the sample correlation coefficient $r_{xy}$ of two random samples $x=\left\{x_{i}\right\}$ and $y=\left\{y_{j}\right\}$ is conventionally defined as $r_{xy}=v_{xy}/(s_{x}s_{y})$, where $v_{xy}$ is their sample covariance and $s_{x}$ and $s_{y}$ are their sample standard deviations, and quantifies the strength and the slope of linear relationships between variable pairs. It is not physically possible to visualize the trivariate and multivariate joint distributions of the estimands.

The pairplot of figure 5 is symmetric with respect to the grid diagonal. We have therefore taken the opportunity to visualize in the same format the bivariate joint distributions of the standard errors of parameter inference $\left\{{\hat{\sigma }}_{k}^{j}\right\}$ corresponding to the estimate of the parameter values k for each cell j. These are plotted in magenta above the main diagonal in figure 5. Univariate distributions of these quantities are not presented. In contrast to the estimands, the standard errors show significant positive linear correlations, as also quantified in figure 6*b*.

#### 3.4.3. Action potential biomarkers as ‘marginal’ functions of parameter estimands

**Figure 7.**
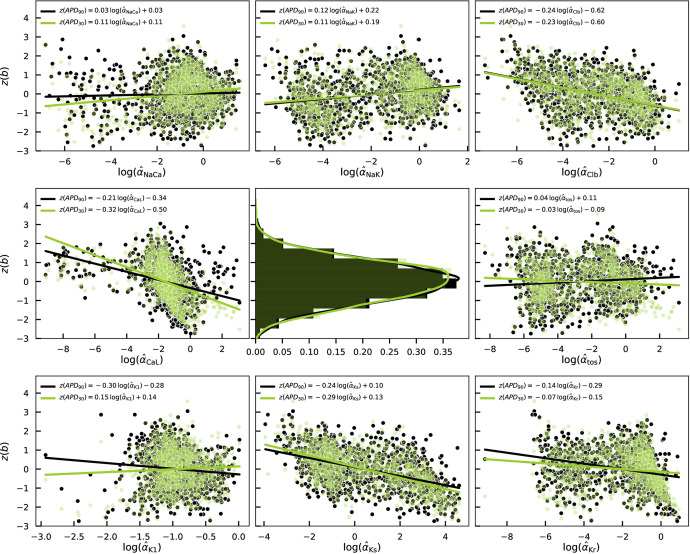
APD_90_ and APD_30_ as ‘marginal’ functions of each Shannon parameter estimand as indicated on the abscissas are shown in the peripheral panels as scatter plots. Simultaneously, all other estimands vary randomly. Univariate linear regression fits for both biomarkers are included throughout and their regression coefficients are stated in the legends of each panel. Normalized histograms and kernel density estimates for the biomarkers are plotted in the central panel. Histograms of the estimands are included in figure 5. In all plots, the $APD_{90}$ biomarker is coloured in black, and the $APD_{30}$ biomarker is shown in yellow-green and made transparent for clarity of visualization. The biomarkers are z-standardized and the estimands are logarithmically transformed.

**Figure 8.**
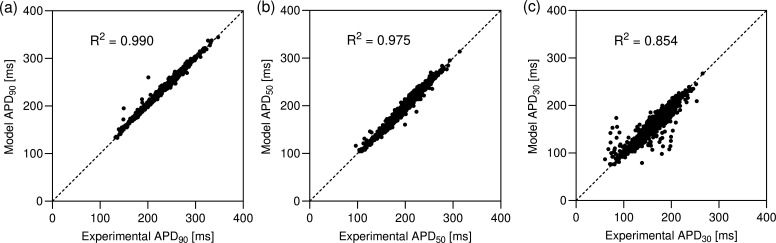
Cell-by-cell comparison of selected biomarkers (APD_90_ in (a), APD_50_ in (b), and APD_30_ in (c)) computed from cell-specific Shannon models (on the ordinates) with their experimental values (on the abscissas). The y=x dashed line denotes the position of perfect agreement. Values for the coefficient of determination $R^{2}$ between the experimental values and model predictions are stated in the legends. The *p*-values from a *t*‐test for statistical significance of the linear relationships are less than 0.001 in all cases.

Mathematical models relate internal parameters to physiological observables, see discussion of figure 2. Figure 7 provides an example of this, now on a population level. Out of the many quantities that may be evaluated using the cell-specific Shannon models in the population, we have chosen to visualize the dependence of APD_90_ and APD_30_ on each of the parameter estimands. APD_90_ and APD_30_ are cellular biomarkers that are commonly measured and reported in the electrophysiology literature. To ensure the values of the two biomarkers are comparable, they have been normalized by their standard z-score function z(b)=(b−μ)/σ, where μ is the mean of a sample population of random values b and σ is its standard deviation. While not unexpected, it is remarkable, that when standardized in this way the distributions of both biomarkers become nearly identical. Similarly to the difficulties in visualizing the eight-variate probability distribution of Shannon parameter estimands, we are restricted to presenting the parameter dependencies of APD_90_ and APD_30_ in one or two dimensions. Thus, to borrow a statistical term, the dependencies shown in figure 7 must be interpreted as ‘marginal’ functions in the sense that, in addition to the parameter dependence that is explicitly plotted, all other parameter estimands also vary simultaneously. Figure 7 reveals that there are no simple functional, and even less so, linear relationships between the two biomarkers and the underlying parameter values. Nevertheless, we have included univariate linear regression fits to the panels in figure 7. Prompted by the lack of simple univariate linear relations, as well as by prior studies in different models [16,43,44], we have also computed multivariate linear regression models of the biomarkers as a function of all eight estimands. The results along with coefficients of determination and a comparison with the univariate regressions are shown in the electronic supplementary material, figure S7 and table S2. The multivariate linear regressions have coefficients of determination $R^{2}\approx 0.6$ and provide better fits than the univariate linear regressions ($R^{2}\approx 0.1$). However, this is only a moderately good fit at best and cannot serve as a replacement for cell-specific Shannon models which provide a near exact match to the experimental biomarker values (e.g. $R^{2}=0.99$ of APD_90_) as shown in figure 8. *p*-values for all fits are listed in the figures and indicate high statistical significance. The cell-by-cell agreement between models and myocytes demonstrated in figure 8 is the key advantage of the constructed cell-specific population compared to earlier attempts to calibrate populations of models such as [3,15,16,37].

The ‘marginal’ functional dependencies of APD_90_ on all pairs of estimands are included in the electronic supplementary material, figure S6 in the form of a grid similar to the pairplot of figure 5. Additional biomarkers, including APD_50_, peak and resting voltage values and AP bulk integral have also been computed for all cells and are provided in the electronic supplementary material, table S5. As expected, there is an excellent agreement between biomarker values determined from the cell-specific models and values measured experimentally for each individual cell as well as on a population level as seen in the electronic supplementary material, figure S8.

#### 3.4.4. Prediction of intracellular calcium biomarkers

The contraction of a cardiac myocyte is triggered by an intracellular rise in calcium concentration. Intracellular calcium concentration was not measured in our experiments. We have predicted the values of four intracellular calcium biomarkers. The electronic supplementary material, table S5 includes the predicted values for all 1180 cells in the population, while table 2 provides sample statistics and ranges for the population. The predicted values of the end diastolic concentration and the peak systolic concentration of [Ca^2+^]_i_ fall within the experimental ranges reported in [45–47]. All predicted biomarkers are also comparable in value to those of the Shannon baseline model, also listed in table 2. The Shannon baseline model is, of course, a synthesis of data and behaviour from a number of experimental studies as detailed within [30]. Other quantities may be evaluated on demand.

**Table 2.** Sample statistics for selected [Ca^2+^]_i_ biomarkers. (The corresponding values for the baseline Shannon model and ranges derived from experimental data with associated references are listed in the last two columns for comparison. The following abbreviations are used: ED, [Ca^2+^]_i_ at the end of diastole; PSV, peak systolic value of [Ca^2+^]_i_; tpeak, time from stimulus to peak [Ca] ${,}_{i}^{2+}$; D50, period of time when [Ca^2+^]_i_ remains elevated above a threshold of 50% recovery from the peak value to the resting value, informally duration at 50% amplitude. Percentages in the column headings denote quartiles of the data.)

biomarker	count	mean	std	min	25%	50%	75%	max	baseline	exp. range and [references]
ED (mM)	1180	1.14e − 4	6.00e − 6	1.00e − 4	1.12e − 4	1.15e − 4	1.17e − 4	1.52e − 4	1.02e − 4	(8e − 5,2.5e − 4) [45,46],
PSV (mM)	1180	5.56e − 4	1.90e − 5	4.52e − 4	5.50e − 4	5.62e − 4	5.69e − 4	5.75e − 4	5.24e − 4	(4.4e − 4, 1.6e − 3) [45–47],
tpeak (ms)	1180	3.06e + 1	1.29e + 0	2.81e + 1	2.98e + 1	3.04e + 1	3.10e + 1	3.76e + 1	3.41e + 1	
D50 (ms)	1180	1.15e + 2	1.46e + 0	1.03e + 2	1.15e + 2	1.15e + 2	1.16e + 2	1.19e + 2	1.15e + 2	

### 3.5. Uncertainty on estimates

Within the limitations of our study, each of the constructed cell-specific models closely matches its corresponding experimental AP waveform as extensively demonstrated in the electronic supplementary material, figure S1. However, we recognize that our estimates are subject to further uncertainties. These include structural, initial condition, simulator/procedural and others uncertainties (see [41]). In lieu of a disclaimer, we illustrate and attempt to quantify some of these uncertainties here.

#### 3.5.1. Initial conditions uncertainty

A limitation of our study is that estimation is performed on single AP waveforms without prepacing. Prepacing is a procedure of forcing equation (2.1) into a stable limit cycle by a periodic stimulus most often in the form of a train of short rectangular impulses. Prepacing mimics experimental and physiological conditions and represents an attempt to avoid the uncertainty about the initial conditions of problem (2.1). While our experimental data are indeed prepaced by 600 APs, it is computationally prohibitive to optimize all cells with prepacing. To assess the effect of this discrepancy, we re-fitted nine typical cells with prepacing by 600 APs. The ratios $\lambda_{i}={\hat{\alpha }}_{i}^{\text{prep}}/{\hat{\alpha }}_{i}$ between estimates obtained with prepacing ${\hat{\alpha }}_{i}^{\text{prep}}$ and those obtained without prepacing ${\hat{\alpha }}_{i}$ are listed in the electronic supplementary material, table S7. Because the nine example cells were selected so as to have AP waveforms ranging from relatively short to relatively long, we assume that they sample the entire parameter space of the problem well, and we compute the following mean ratios:


(3.3) λNaCa=1.07e+00,     λNaK=2.48e-01,     λClb=5.19e-01,     λCaL=8.22e-01,     λtos=3.20e+01,     λK1=1.07e+00,     λKs=2.00e-01,     λKr=2.87e+00.     

As a first approximation, the parameter estimates without prepacing (plotted in figure 5, listed in the electronic supplementary material, table S3 and discussed throughout the text) can be converted to ‘prepaced’ estimates by multiplying them with the ratios of equation (3.3), so for any cell α^iprep=λiα^i for i∈{NaCa, NaK, …, Kr}.

#### 3.5.2. Procedural uncertainty

The mapping of fluorescence intensity to electric potential values is justified but somewhat arbitrary. To assess the effect of this choice, we have re-fitted 125 times cell uid: 21051_run1cell16 while mapping the values of $V_{\text{rest}}$ and $V_{\text{plateau}}$ to random values sampled from normal distributions with standard deviations of 1 mV from the means of −86 and 0 mV, respectively. Sample statistics for the resulting estimates are given in the electronic supplementary material, table S8. The mean values of the estimates remain close to those found when rest and plateau voltages are mapped to −86 and 0 mV, respectively.

#### 3.5.3. Structural uncertainty

The Shannon [30] model is an oversimplification of a real rabbit ventricular myocyte. Indeed, ion channel structures and kinetics are still under study. Currents can be modelled by various alternative approximations, e.g. Ohmic, Goldman-Hodgkin-Katz, Markovian. Intracellular processes may be described by lumped-compartment or spatially extended sub-models, etc. To quantify such structural uncertainty, one may estimate parameters of an alternative model. In the present case, such exercise is of limited validity because the alternative detailed rabbit ventricular myocyte model, that of Mahajan *et al*. [48], is a direct extension of [30].

In the light of this discussion, no claim is made about the uniqueness of our estimates.

## 4. Conclusion

### 4.1. Summary

Advances in optics-based techniques for cardiac electrophysiology have made it possible to develop high-throughput platforms capable of recording transmembrane voltage from several thousand uncoupled cardiomyocytes per hour [24]. Recent experiments based on these techniques reveal significant heterogeneity in uncoupled healthy myocytes both between hearts as well as from identical regions within a single heart [29]. By contrast, the mathematical modelling of electrophysiological variability lags behind. Models of cardiomyocyte APs describe generic cell archetypes and do not capture inter-cell variability. This makes them ill-suited for direct use as digital twins or for safety-related applications such as pharmaceutical drug discovery and toxicity assessment. To address this issue, we created a population of nearly 1200 individualized cell-specific mathematical models capable of reproducing transmembrane potentials experimentally measured from healthy rabbit ventricular myocytes. We started from the model of Shannon *et al*. [30], a well-regarded and detailed mathematical model of the ionic currents in a generic rabbit ventricular myocyte. We selected eight of the parameters of the model as ones most likely to affect the AP shape following [29]. We estimated cell-specific values of the eight selected parameters by fitting voltage values computed from the Shannon model to the noisy experimental trace from each of the biological cells. We assumed that errors in the experimental measurements were normally distributed about the true signal. We formulated a corresponding likelihood function to measure the probability of obtaining specific experimental measurements at particular parameter values. We then invoked the maximum likelihood principle to find point estimates of the parameters of interest, measured the standard errors of estimation and quantified the overall goodness-of-fit. We used the CMA-ES, a gradient-free random search algorithm [49], to find a global maximum of the likelihood. We validated the methodology by re-fitting synthetic data precomputed at known parameter values and then described in detail the fitting of nine typical experimental measurements. We also tested the approach by performing Bayesian inference which allowed us to assess the uniqueness of estimates and the size of the estimation errors. We proceeded to apply the approach to AP waveforms recorded from 1228 rabbit ventricular myocytes using a voltage-sensitive fluorescent indicator. We accepted 1180 fits as sufficiently good and thus obtained a large population of cell-specific Shannon models where each model reproduces accurately the measured electrophysiological response of an individual cell. We interpreted this population as a random sample from the phenotype of normal healthy rabbit myocytes. We then attempted to characterize the probability distribution of the phenotype by calculating basic summary statistics and visualizing all univariate and bivariate marginal distributions for the constructed sample of parameter estimates. A population of cell-specific mathematical models may have a large number of diverse applications. As a simple demonstration, we computed ionic current densities for a small subset of cells, as well as a number of biomarkers commonly measured in experiments, including APD_30_ and APD_90_ and revealed their dependencies on the internal state of the cells as quantified by their Shannon model parameters.

### 4.2. Headline results

In comparison with earlier studies that investigate cellular electrophysiological variability by calibrating populations of models and applying parameter identification techniques, the cell-specific Shannon models reported here not only match experimentally measured biomarker ranges and distributions on a population level, but also replicate experimental biomarker values on a cell-by-model basis. Our work confirms that it is possible to efficiently and accurately estimate model parameters at scale. We find that model parameter distributions vary over large ranges and that parameter values are weakly inter-correlated. As a result, high-level summary observables such as APD_90_, do not depend strongly on any one particular cellular property of the myocyte or associated mathematical model parameter.

### 4.3. Limitations, extensions and future directions

The methodology and the applications presented here can be extended and refined in a number of directions. A current limitation of our study is that optimization is performed on single AP waveforms, as it is computationally prohibitive to optimize for trains of paced APs. The study is restricted to stimulation at 2 Hz and the AP dynamics at other pacing rates was not studied. Bi-phasic stimuli will be considered in future refinements of the study to better approximate the field stimulation protocol used in experiments. The parameter $E_{\text{Na,SL}}$ can be included in the optimization to better reflect the lack of pronounced spikes in the experimental waveforms. Further investigation is required into the choice and number of fitting parameters. We are presently undertaking a global sensitivity analysis of the Shannon model to establish a formal order of its most sensitive parameters. Ideally, it is desirable to fit all of the nearly two hundred parameters of this model. While all accepted fits accurately reproduce their corresponding experimental measurement, it is not certain that the estimated parameter values are the only possible ones. Generally, the parameter estimation problem lacks a unique solution, but closer-to-reality estimates can be achieved by incorporating supplementary data. Examples include complex pacing protocols, experimental APD restitution assessment, voltage-clamp measurements, simultaneous calcium transient recordings via microfluorimetry or applying selective ion channel modulators such as E4031 for hERG (Human Ether-à-go-go-Related Gene) and benzamil for NCX (Sodium-Calcium Exchanger). This approach is exemplified by Zhang *et al*. [50] who demonstrated feasibility using two separate APs per cell. Alternative models of the AP exist for most generic cell types, including e.g. the model of Mahajan *et al*. [48] for the rabbit ventricular myocyte. It is straightforward to adapt our parameter estimation procedure for alternative models and appropriate model selection criteria should be investigated. From a technical viewpoint, a large number of alternative optimization methods exist, including both gradient-descent and random search methods. Whether these modifications will result in increased accuracy and efficiency, and whether this level of detail is needed on a population level, respectively, should be avenues for further study. From an electrophysiology viewpoint, it will be important to constrain and/or extend the inference procedure by additional experimental measurements. For example, measurements of myocyte contraction may be incorporated by coupling the AP model to an appropriate model of cell contractility as performed by Huethorst *et al.* [51]. Machine learning methods of the type developed in [52] can be combined with the parameter probability distributions constructed here to identify the underlying electrophysiology of various cell sub-populations. Another direction, that we plan to follow almost immediately, is to study the pharmacodynamics of anti-arrhythmic drugs. We have performed measurements of the response of all cells reported in this work under the action of various concentrations of dofetilide. Paired AP waveforms before and after drug administration are available for each cell. These will be used to infer dofetilide pharmacodynamics assuming the internal state of the myocyte can be accurately determined by the methodology developed here or alternatively to further constrain the parameter estimation procedure assuming dofetilide pharmacodynamics is well-known. This and equivalent data for a different ion channel blocker will be part of a comprehensive examination of the action of drugs that affect repolarization and the subject of a future publication. Possibilities for further applications are numerous, and we invite the readers to make use of the open source software [53] provided with this work and conduct their own investigations.

## Data Availability

Codes and data are available from [53]. Supplementary material is available online [54].
